# Quetiapine ameliorates sensorimotor gating, recognition memory, and neuroendocrine plasticity in chronic stress–induced female rats

**DOI:** 10.1007/s11011-026-01834-8

**Published:** 2026-03-28

**Authors:** Burcu Çevreli, Öznur Özge Özcan, Kübra Kayıkçı, Elif İrem Başıeğri

**Affiliations:** 1 Department of Physiology, Faculty of Medicine, Üsküdar University, İstanbul, Turkey; 2 Neuropsychopharmacology Practice and Research Center, Üsküdar University, Istanbul, Turkey; 3 Molecular Biology and genetics, Faculty of Engineering and Natural Sciences, Üsküdar University, Istanbul, Turkey; 4 Department of Medical Biochemistry, Faculty of Medicine, Üsküdar University, Istanbul, Turkey; 5 Department of Medical Biochemistry, Institute of Health Sciences, Istanbul University, Istanbul, Turkey

**Keywords:** Acoustic Startle Response, BDNF, Corticosterone, Female Rats, Novel Object Recognition (NOR), Prepulse Inhibition (PPI), Quetiapine, Recognition Memory, Sensorimotor Gating, Unpredictable Chronic Mild Stress (UCMS)

## Abstract

Chronic stress induces sensorimotor, cognitive, and neuroendocrine alterations, particularly in females who exhibit heightened vulnerability to stress-related disorders. This study tested the hypothesis that chronic quetiapine administration during ongoing unpredictable chronic mild stress (UCMS) would attenuate stress-induced impairments in sensorimotor gating, recognition memory, and HPA-axis–related biochemical markers in female rats. Adult female Wistar rats were exposed to a 9-week UCMS paradigm, with quetiapine (10 mg/kg/day, i.p.) administered during the final 3 weeks. Behavioral outcomes were assessed using prepulse inhibition (PPI), startle reactivity, and the Novel Object Recognition (NOR) test. Serum and hippocampal corticosterone and BDNF levels were quantified by ELISA. Chronic stress significantly reduced PPI and recognition memory performance and increased serum and hippocampal corticosterone levels. Quetiapine treatment improved PPI and startle responsiveness, restored NOR discrimination index values, and partially attenuated stress-induced corticosterone elevations. Hippocampal BDNF levels were elevated in stressed animals and were modulated toward intermediate levels following quetiapine treatment. These findings indicate that chronic quetiapine administration mitigates behavioral and neuroendocrine alterations induced by prolonged stress in female rats.

**Graphical abstract:**

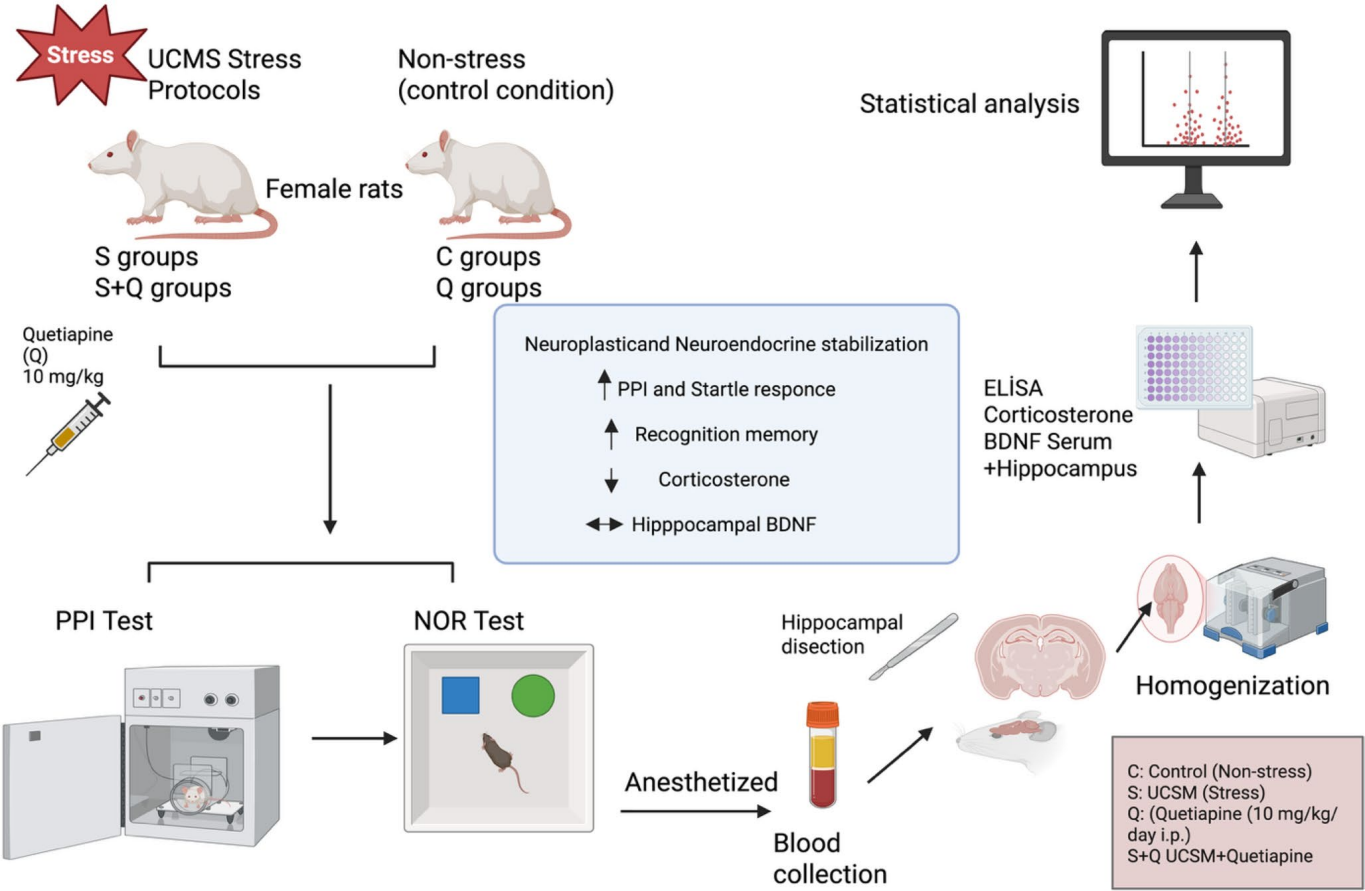

**Supplementary Information:**

The online version contains supplementary material available at 10.1007/s11011-026-01834-8.

## Introduction

Stress-related affective disorders represent some of the most prevalent and debilitating psychiatric conditions worldwide, contributing substantially to global disease burden and disability. Chronic stress exposure is recognized as a principal environmental factor in the pathophysiology of depression, exerting profound effects on hypothalamic–pituitary–adrenal (HPA) axis regulation, neuroplasticity, and limbic and induce neuroinflammatory changes within limbic regions such as the hippocampus circuitry (Willner, 2017; Duman & Monteggia, 2006). Prolonged activation of glucocorticoid signaling has been shown to decrease neuronal survival, synaptic remodeling, and neurogenesis—key processes that underlie cognitive and emotional regulation (Willner, 2017; Nollet et al., 2021). Consequently, animal models replicating chronic stress exposure, such as the Unpredictable Chronic Mild Stress (UCMS) paradigm, have become indispensable tools in elucidating the neurobiological underpinnings of depression and testing novel therapeutic strategies (Seewoo et al., 2020). Among the neural substrates affected by chronic stress, the hippocampus is particularly vulnerable. Structural and functional impairments in the hippocampus have been consistently observed in both human patients with depression and preclinical models of chronic stress (Kim et al., 2015). Decreased levels of brain-derived neurotrophic factor (BDNF)—a key modulator of synaptic plasticity, neuronal survival, and cognitive processing—are among the most reproducible biological correlates of depressive pathology (Duman & Monteggia, 2006; Toader et al., 2025). BDNF deficiency has been linked to impaired hippocampal-dependent learning and memory, as well as reduced responsiveness to conventional antidepressant treatment. Notably, antidepressant interventions, including selective serotonin reuptake inhibitors and atypical antipsychotics, can restore hippocampal BDNF expression and promote neurogenesis, suggesting that enhancement of BDNF signaling is a critical mechanism for therapeutic recovery (Lan et al., 2025). Activation of PKA-CREB-BDNF signaling appears to be a convergent adaptive mechanism across different stress-resilience interventions, as shown by p-Coumaric acid reversing chronic stress–induced neurobehavioral impairments in mice (Cao et al., 2024).

Numerous preclinical and clinical studies consistently report that chronic stress disproportionately affects females, leading to more pronounced impairments in cognition, sensorimotor gating, and stress-related symptomatology (Franceschelli et al., 2014; Helpman et al., 2023; Hillerer et al., 2019; Klinger et al., 2019; Mancini et al., 2025; Xia et al., 2023). Moreover, stress-related psychiatric disorders such as anxiety and depression show higher prevalence and severity in women, emphasizing the need for female-focused preclinical models (Shawon et al., 2024; Senserrich et al., 2025). Recent human evidence also shows that stress-related cortisol reactivity interacts with gender to amplify psychological distress, further supporting the heightened stress vulnerability observed in females (Chong et al., 2025). Despite women representing a majority of depression cases globally, female rodents remain underrepresented in preclinical psychopharmacology studies (Sramek et al., 2016; Tucker et al., 2022).

Quetiapine, an atypical antipsychotic widely used in the treatment of schizophrenia and bipolar depression, has gained increasing attention for its antidepressant and neuroprotective properties. Beyond dopamine and serotonin receptor modulation, quetiapine exerts profound effects on neuroplasticity pathways, oxidative stress regulation, and neurotrophin expression (Yılmaz et al., 2013). Experimental evidence indicates that quetiapine enhances hippocampal BDNF levels and reverses stress-induced neuronal atrophy, thereby ameliorating cognitive deficits associated with chronic stress. Its active metabolite, norquetiapine, additionally modulates norepinephrine reuptake and α₂-adrenergic receptors, mechanisms that may synergistically contribute to antidepressant efficacy. Importantly, quetiapine’s favorable influence on memory, learning, and stress reactivity has been observed in both clinical populations and preclinical stress models, yet data regarding its chronic effects in female rodents, who exhibit distinct neuroendocrine responses to stress, remain scarce. Investigating the interaction between quetiapine and the stress axis in females may thus provide valuable insight into its potential to restore neuroplastic and cognitive integrity under chronic stress conditions (Komanovalı et al., 2025; Willner, 2017; Seewoo et al., 2020).

The Novel Object Recognition (NOR) task, which relies on intact hippocampal and perirhinal cortex function, is a sensitive measure of recognition memory and thus an ideal behavioral index to assess stress-induced cognitive decline and its pharmacological modulation. In addition to hippocampal-dependent recognition memory, sensorimotor gating represents another critical neurophysiological mechanism affected by chronic stress and psychiatric disorders. Prepulse inhibition (PPI) of the acoustic startle reflex is a well-established operational measure of sensorimotor gating, reflecting the brain’s ability to filter out irrelevant stimuli (Braff & Geyer, 1990). Consistent with these observations, recent findings indicate that even acute stress exposure is sufficient to reduce PPI in both male and female Wistar rats, further supporting the sensitivity of sensorimotor gating circuits to stress-related disruption (Santos-Carrasco et al., 2025). Deficits in PPI are consistently observed in patients with schizophrenia, bipolar disorder, and major depression, and are associated with dysregulated dopaminergic, serotonergic, and glutamatergic signaling (Brymer et al., 2021; Geyer et al., 2001). Chronic stress exposure has been shown to reduce PPI performance in rodents, paralleling the sensory gating abnormalities found in clinical populations (Swerdlow et al., 2008). Importantly, atypical antipsychotics such as quetiapine have demonstrated the capacity to reverse stress- or drug-induced PPI impairments, suggesting a normalization of subcortical sensorimotor processing and improved prefrontal inhibitory control (Özcan et al., 2024). Therefore, integrating PPI testing into the current experimental framework allows for the assessment of both cognitive (NOR) and neurophysiological (PPI) dimensions of quetiapine’s antidepressant-like actions under chronic stress conditions.

In our previous study, we demonstrated that quetiapine exerted clear therapeutic effects when administered after the completion of chronic stress exposure, reversing anxiety- and depression-like behaviors induced by the CUMS paradigm in female rats (Komanovalı et al., 2025). However, the neurobehavioral and neuroendocrine consequences of initiating quetiapine treatment while stress exposure is still ongoing remain largely unexplored, particularly in females who show heightened vulnerability to chronic stress–related cognitive and sensorimotor disturbances. Building upon our earlier findings, the present study investigated whether chronic quetiapine administration delivered during continued UCMS exposure can mitigate stress-induced impairments in sensorimotor gating, recognition memory, and HPA axis function. By integrating PPI and startle reactivity measures with Novel Object Recognition performance and quantification of serum and hippocampal BDNF and corticosterone levels, this work aims to elucidate the extent to which quetiapine modulates neuroplastic, cognitive, and endocrine pathways under persistent stress conditions. Specifically, we tested whether concurrent quetiapine administration during ongoing UCMS exposure attenuates stress-induced impairments in sensorimotor gating and recognition memory, and whether these behavioral effects are paralleled by coordinated modulation of corticosterone and hippocampal BDNF levels. By examining these endpoints within the same experimental framework, the study allows differentiation between stress-induced alterations and pharmacological modulation under continued stress exposure.

## Materials and methods

### Experimental design and animal groups

Adult female Wistar Albino rats (8–10 weeks old, 250–300 g) were obtained from an accredited breeding facility and housed in groups of three per cage under controlled environmental conditions (22 ± 2 °C; 12:12 h light/dark cycle; lights on at 07:00 a.m.). Standard pellet chow and water were available ad libitum except during stress procedures. All experimental procedures were approved by the Institutional Animal Care and Use Committee and conducted in accordance with the EU Directive 2010/63/EU for animal experimentation. All experimental procedures involving animals were conducted in accordance with institutional and national guidelines for the care and use of laboratory animals. Ethical approval for this study was obtained from the Animal Research Ethics Committee. All experimental procedures were performed in the controlled laboratory environment of the Research Center. The study was designed using only female rats in order to specifically investigate stress-related neurobehavioral and neuroendocrine alterations in a sex that exhibits heightened vulnerability to affective disorders. This selection was based on accumulating preclinical and clinical evidence demonstrating increased stress sensitivity and higher prevalence of mood disorders in females.

### G*Power sample size calculation

A priori sample size estimation was conducted using G*Power 3.1 to ensure adequate statistical power for detecting group differences in the primary behavioral outcomes (PPI and NOR discrimination index). Based on prior rodent studies reporting medium-to-large effect sizes for chronic stress and quetiapine interventions (f = 0.40–0.45), a one-way ANOVA with four groups, an alpha level of 0.05, and a desired power of 0.80 indicated a minimum requirement of 8 animals per group (Faul et al., 2007). To account for potential attrition associated with chronic stress exposure and behavioral testing, the group size was increased to *n* = 10, yielding a total sample of 40 rats. This sample size provides sufficient power to detect stress-by-treatment interactions across behavioral, endocrine, and neuroplasticity-related outcomes.

A total of 40 adult female Wistar rats (8–10 weeks old) were randomly assigned to four groups (*n* = 10 per group): Control (C), UCMS (S), UCMS + Quetiapine (S + Q), and Quetiapine-only (Q). All stressed animals (S and S + Q groups) and non-stressed animals (Control and Q groups) were housed in two separate experimental rooms to prevent any sensory or behavioral cross-exposure. Estrous cycle monitoring was not performed during the experimental procedures. This decision was made to avoid additional handling- and sampling-related stress that could interfere with the chronic unpredictable mild stress (UCMS) paradigm, behavioral testing, and neuroendocrine measurements. Animals were randomly assigned to experimental groups, and all behavioral assessments and tissue collections were conducted across different estrous phases in an unbiased manner. Given the prolonged duration of the UCMS protocol and chronic quetiapine administration, the study was designed to capture integrated stress-related behavioral and neurobiological outcomes rather than estrous phase–specific effects.

The C group was maintained under standard laboratory conditions and, similar to the quetiapine-treated groups, received daily intraperitoneal injections of 0.9% saline for 21 days as the vehicle control, without exposure to stress or pharmacological treatment. The S group was exposed to the UCMS protocol for nine consecutive weeks without receiving any drug treatment. The S + Q group was subjected to the same 9-week UCMS procedure followed by intraperitoneal (i.p.) administration of quetiapine fumarate (10 mg/kg/day) for 21 consecutive days. The Q group received quetiapine fumarate at the same dose and duration (10 mg/kg/day, i.p., 21 days) under normal housing conditions without exposure to stress. Quetiapine solutions were freshly prepared in sterile saline and administered once daily at the same hour each day to minimize circadian variation. All pharmacological administrations were performed in the light phase between 09:00 and 10:00 a.m. to control for circadian influences on drug pharmacokinetics, behavioral performance, and neuroendocrine measures.

**Fig. 1:**
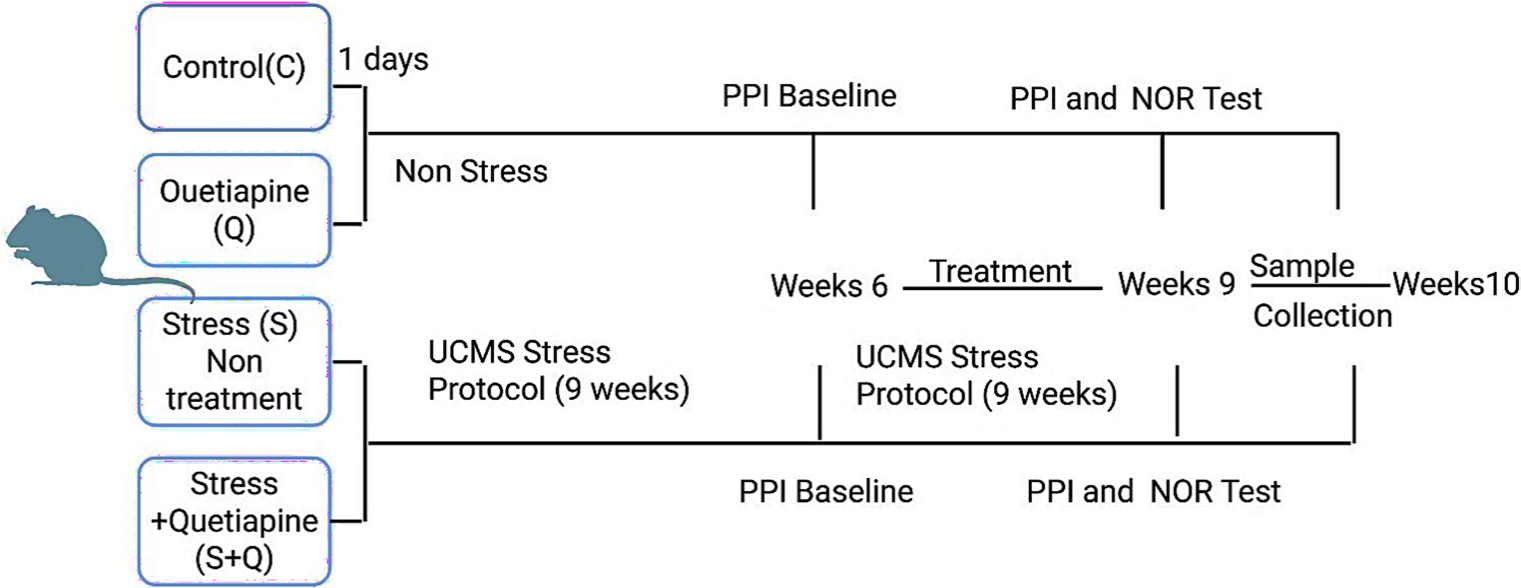
A timeline diagram for experimental design

The Fig. 1 illustrates the 10-week experimental timeline for the four study groups. The UCMS protocol was applied throughout 9 weeks for both stress-exposed groups. At Week 6, all animals underwent a baseline PPI session that served as an auditory screening procedure. This baseline assessment was performed to confirm adequate auditory startle responsiveness, as rats exhibiting a startle amplitude more than 20 dB below the expected threshold are typically excluded from subsequent PPI-based paradigms due to potential hearing impairment. In the present study, none of the animals showed reduced startle reactivity, and therefore no exclusions were made and the study proceeded with the full cohort.

Quetiapine treatment was initiated at Week 6 for animals in the Q and S + Q groups and continued until Week 9, coinciding with ongoing UCMS exposure in the S + Q group. On days when pharmacological treatment coincided with UCMS procedures, quetiapine was administered at least 60 min prior to stress exposure to avoid acute stress–drug interaction effects and to ensure stable systemic drug availability during stress induction.

During Week 9, all rats completed the second behavioral test battery, consisting of PPI and the Novel Object Recognition (NOR) test, to evaluate stress- and treatment-related alterations in sensorimotor gating and recognition memory. At Week 10, blood and tissue samples were collected from all animals for subsequent endocrine, biochemical, and molecular analyses.

### Unpredictable chronic mild stress (UCMS) protocol- 9 weeks

To establish a chronic stress phenotype, adult female Wistar rats were subjected to a 9-week Unpredictable Chronic Mild Stress (UCMS) paradigm, adapted from Willner (1997, 2017) and standardized according to Nollet et al. (2021) and Seewoo et al. (2020). This model is widely recognized for its translational validity in mimicking depressive-like behaviors through chronic and unpredictable environmental stressors.

#### Experimental design and general conditions

All animals were housed individually in standard polycarbonate cages (42 × 26 × 15 cm) under controlled environmental conditions (temperature: 22 ± 1 °C; humidity: 55 ± 10%; 12:12 h light/dark cycle, lights on at 07:00 a.m.). Standard laboratory chow and water were available ad libitum except during specific stress procedures. Animals were allowed a 7-day acclimatization period prior to the UCMS procedure (Willner, 1997).

#### Stress Paradigm

The UCMS regimen consisted of exposure to mild, non-painful stressors applied once or twice daily in a pseudorandom order to minimize predictability and prevent habituation. Each stressor was separated by at least 12 h, and combinations were varied across the 9-week schedule. The specific stressors included:

##### Social stress

Rats were placed in an empty cage previously inhabited by another animal for 30 min before being returned to their home cage.

##### Bedding change

Cage bedding was replaced 2–6 times within 24 h, including soiled bedding from control cages.

##### No-bedding exposure

All bedding was removed for 1–6 h.

##### Wet bedding

Approximately 125 mL of tap water was added to the cage bedding for 1–6 h to create a damp environment.

##### Water stress

Bedding was removed and replaced by 125 mL of water (20 °C) to generate a shallow 1 cm layer for 15–30 min.

##### Cage tilt

Cages were tilted backward at a 45° angle for 1–4 h.

##### Fecal exposure

About 60 mL of feces-containing bedding from other rats was added to the cage for 1–2 h.

##### Restraint stress

Animals were restrained in ventilated Plexiglas tubes (6.5 cm × 3.7 cm) for 15–30 min.

##### Circadian disruption

Light/dark cycles were altered (e.g., reversal or division into 6-h intervals) and occasionally interrupted by short light exposures (30 min–2 h) during the dark phase.

These stressors have been validated for inducing behavioral and physiological correlates of depression, including anhedonia, decreased exploratory behavior, and HPA axis dysregulation (Willner, 2017; Nollet et al., 2021).

### Drug administration

Pure QET (hemifumarate) was used in this study (Cayman Chemical, USA; CAS Number: 111974-72-2). After the UCMS induction period, rats received either vehicle (0.9% saline) or quetiapine fumarate (10 mg/kg/day, intraperitoneal) dissolved in 0.9% saline solution for 21 consecutive days. Dosing was based on prior preclinical studies demonstrating antidepressant-like effects and hippocampal neuroprotection (Lan et al., 2025; Yılmaz et al., 2013).

### Prepulse inhibition (PPI) measurement method

The measurement of prepulse inhibition (PPI) of the acoustic startle reflex was performed using an automated startle response system (an automated startle response system; SR-LAB Startle Reflex System, Model: SR-LAB-STARTLE, San Diego Instruments, San Diego, CA, USA; RRID: SCR_015785). Prior to behavioral testing, all rats were gradually acclimated to handling for three consecutive days to reduce stress-related variability. On the fourth day, each animal was placed in the startle chamber for a 15-minute habituation session under constant background noise (70 dB) without any auditory stimuli. Baseline startle responses were recorded on the fifth day in the absence of pharmacological intervention. Animals exhibiting a mean startle amplitude below 20 arbitrary units were excluded from subsequent analysis. Testing sessions began with a 5-minute acclimation period followed by a sequence of five initial 120 dB startle pulses to stabilize the startle response. Each experimental session consisted of ten randomized stimulus blocks presented at variable inter-trial intervals (10–30 s). Within each block, the following five stimulus types were presented in pseudorandom order:

1. A 120 dB acoustic pulse (40 ms duration).
2. A 20 ms prepulse at + 4 dB above background (74 dB), followed by a 120 dB pulse (40 ms) after a 100 ms delay.
3. A 20 ms prepulse at + 8 dB above background (78 dB), followed by a 120 dB pulse (40 ms) after a 100 ms delay.
4. A 20 ms prepulse at + 16 dB above background (86 dB), followed by a 120 dB pulse (40 ms) after a 100 ms delay.

5. Background noise only (70 dB), used to monitor baseline movement within the chamber.

Each session lasted approximately 25 min. Between trials, chambers were cleaned with 70% ethanol to eliminate odor cues. Testing sessions were separated by at least 10 days to minimize habituation effects. Higher PPI values indicate stronger sensorimotor gating capacity. All measurements were conducted under standardized environmental conditions (temperature 22 ± 2 °C, relative humidity 50 ± 5%, 12 h light/dark cycle) (Özcan et al., 2024). The percentage of inhibition produced by each prepulse intensity was calculated using the following formula:

$$\%PPI=\left(1-\frac{Mean\;Startle\;Amplitude\;with\;Prepulse}{Mean\;Startle\;Amplitude\;without\;Prepulse}\right)\times100$$

All PPI testing sessions were conducted during the light phase between 10:00 and 13:00 h, and testing order was kept constant across groups to minimize circadian and order-related variability.

### Novel object recognition (NOR) test

Recognition memory was evaluated using the Novel Object Recognition (NOR) task with a 24-h retention interval. The test apparatus consisted of a square open-field box (60 × 60 × 40 cm) made of opaque Plexiglas. All sessions were conducted under dim light (≈ 30 lx) and recorded by an overhead camera.

#### Habituation phase

Each rat was allowed to freely explore the empty arena for 10 min per day for two consecutive days to minimize novelty-related anxiety.

#### Training phase

On day 3, two identical objects were placed symmetrically in the arena. Each rat was placed in the center and allowed to explore both objects for 5 min. Object exploration was defined as the animal directing its nose toward the object at a distance ≤ 1 cm or touching/sniffing it, excluding climbing or sitting on the object.

#### Retention phase (24 h)

After training, rats were returned to their home cages for 24 h without exposure to the test apparatus.

#### Testing phase

On the following day, one of the familiar objects was replaced by a novel object of different shape and texture. Each rat was again allowed to explore for 5 min. Exploration times for the novel and familiar objects were measured by a blinded observer.

#### Discrimination index (DI)

DI = (Tnovel - Tfamiliar) / (Tnovel + Tfamiliar), where Tnovel and Tfamiliar are the total exploration times for the novel and familiar objects, respectively. A positive DI indicates preference for the novel object, reflecting intact recognition memory. All NOR procedures (habituation, training, and testing phases) were conducted during the light phase between 10:00 and 14:00 h under consistent environmental conditions.

### Sample collection and tissue processing

The day after the behavioral experiments end, animals were deeply anesthetized prior to biological sample collection. Deep Anesthesia was induced with 4–5% isoflurane and maintained at 1.5–2% in oxygen, in accordance with established protocols for terminal procedures in rodent neuroendocrine studies. Adequate anesthetic depth was verified by the absence of corneal and pedal withdrawal reflexes (Oh et al., 2024). Approximately 4–6 mL blood samples were collected from rats via cardiac puncture under anesthesia and transferred into plain (anticoagulant-free) collection tubes (Li et al., 2025). The samples were allowed to clot at room temperature for 20–30 min in an upright position. Following complete coagulation, the tubes were centrifuged at 3000 rpm (approximately 1000–1500×g) for 10–15 min at 4 °C. After centrifugation, the clear supernatant (serum) was carefully aspirated without disturbing the clot or cellular layer and transferred into sterile, labeled microcentrifuge tubes. All sample collection procedures were conducted during the light phase between 09:30 and 10:30 a.m. to control for circadian influences on corticosterone and BDNF levels (Choe et al., 2024).

The whole brain was rapidly removed, and both hippocampi were dissected on an ice-cold glass plate under continuous cold-chain conditions to preserve molecular integrity. The hippocampal region included the hippocampal formation and parahippocampal region, as anatomically defined by Kjonigsen et al. (2015). The hippocampal formation, a C-shaped structure located posteriorly within both cerebral hemispheres, was isolated for biochemical analyses. Each hippocampus was weighed and immediately homogenized without prior freezing in KCl–KH₂PO₄ buffer (12 mM KCl, 0.038 mM KH₂PO₄, pH 7.4) supplemented with a protease inhibitor cocktail (Sigma-Aldrich, USA; Catalog No. P8340). The homogenates were centrifuged at 12,000 × g for 20 min at 4 °C, and the resulting supernatants were collected for subsequent ELISA assays.

### ELISA quantification

The concentrations of BDNF and corticosterone (CORT) were determined in both serum and hippocampal supernatants using rat-specific enzyme-linked immunosorbent assay (ELISA) kits (BT-Lab, Shanghai, China; Rat BDNF ELISA Kit, Catalog No: E-EL-R0010; Rat Corticosterone (CORT) ELISA Kit, Catalog No: E-EL-0160). All analyses were performed according to the manufacturer’s instructions. Absorbance was measured at 450 nm using a microplate reader (Multiskan™ GO Microplate Spectrophotometer, Catalog No: 51119300, Thermo Fisher Scientific, USA). Concentrations were calculated from standard curves generated for each assay. Hippocampal values were normalized to total protein content and expressed as pg/mg protein, whereas serum concentrations were expressed as pg/mL. Hippocampal corticosterone levels were measured to assess region-specific glucocorticoid exposure in a stress-sensitive brain area involved in HPA axis regulation and neuroplasticity. Unlike circulating levels, tissue corticosterone may better reflect local glucocorticoid signaling; therefore, hippocampal measurements were included to complement serum data and to characterize stress-related neuroendocrine changes at the tissue level.

### Statistical analysis

All statistical analyses were performed using GraphPad Prism version 10.0.6. Prior to hypothesis testing, the distribution of each dataset was assessed using the Shapiro–Wilk normality test. For all behavioral (PPI, startle amplitude, discrimination index) and biochemical (serum and hippocampal corticosterone and BDNF) variables, variance homogeneity was evaluated using Brown–Forsythe and Bartlett’s tests to confirm the suitability of parametric analyses. PPI across the three prepulse intensities (+ 4, + 8, +16 dB) was analyzed using a two-way ANOVA with group (C, S, Q, S + Q) and prepulse intensity as factors. When significant main effects or group × intensity interactions were detected, Tukey’s multiple comparison test was applied. Average PPI values and startle amplitude were analyzed using one-way ANOVA followed by Fisher’s LSD post hoc tests, consistent with established PPI methodology. All PPI and startle measures are presented as mean ± SEM. NORT discrimination index values were analyzed using one-way ANOVA followed by Fisher’s LSD post hoc comparisons. Homogeneity of variance criteria were met for all NORT datasets, supporting the use of parametric testing. NORT values are presented as mean ± SEM. Serum and hippocampal corticosterone and BDNF concentrations were analyzed independently using one-way ANOVA. When overall group effects reached significance, Fisher’s LSD post hoc tests were conducted to identify pairwise differences. For datasets displaying unequal variances (e.g., hippocampal BDNF), ANOVA was retained due to its robustness under equal sample sizes (*n* = 10 per group). All biochemical variables are presented as mean ± SD, consistent with graphical representation. Effect sizes (η²) are reported for all ANOVA models to indicate the magnitude of group effects. Statistical significance was defined as *p* < 0.05 for all comparisons. All tests were two-tailed.

## Results

### Effects of stress and quetiapine on sensorimotor gating

**Fig. 2:**
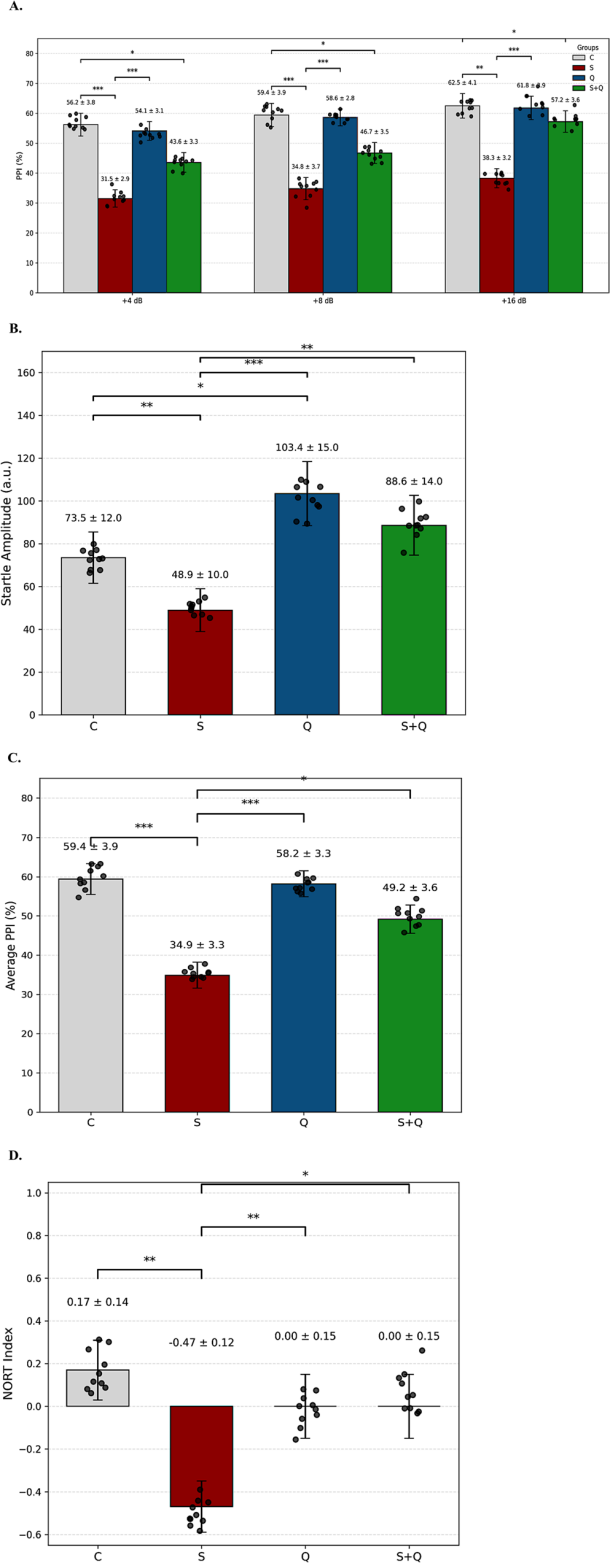
Effects of chronic stress and quetiapine on sensorimotor gating, startle responsiveness, and recognition memory. Statistical comparisons include both Stress (S) vs. Control (C) and Stress (S) vs. Stress + Quetiapine (S + Q) contrasts, which are explicitly indicated on the graphs with horizontal comparison bars. These S vs. S + Q comparisons represent the primary test of quetiapine’s corrective effect under ongoing stress exposure. **(A)** Prepulse inhibition (PPI, %) measured at + 4, +8, and + 16 dB. Chronic stress significantly reduced PPI compared with controls. Quetiapine significantly increased PPI in stressed animals, demonstrating partial-to-substantial recovery depending on prepulse intensity. **(B)** Startle amplitude. Chronic stress reduced baseline startle responsiveness. Quetiapine significantly increased startle amplitude relative to stressed animals, indicating functional improvement. **(C)** Average PPI across intensities. Stress reduced overall gating capacity, whereas quetiapine significantly improved PPI compared with the Stress group, though values remained numerically below controls. **(D)** Novel Object Recognition discrimination index. Chronic stress impaired recognition memory. Quetiapine significantly improved performance relative to the Stress group, reflecting partial restoration of cognitive function. Data are presented as mean ± SEM (behavioral measures). *p < 0.05, **p < 0.01, ***p < 0.001. All S vs. S + Q comparisons are explicitly displayed on the graphs. Detailed descriptive statistics and key p-values for all behavioral outcomes, including PPI, startle amplitude, and NORT discrimination index, are provided in Supplementary Table S2

Two-way ANOVA revealed significant main effects of group (F(3,84) = 6.87, *p* < 0.001, η²=0.24) and prepulse intensity (F(2,84) = 11.93, *p* < 0.0001, η²=0.31), as well as a significant group × intensity interaction (F(6,84) = 3.18, *p* = 0.009, η²=0.12). The assumption of homogeneity of variances was verified using both Brown–Forsythe and Bartlett’s tests (all *p* > 0.78), confirming equal variance across groups. Across all intensities, mean (± SEM) PPI values differed robustly between groups, indicating a clear stress-induced disruption in sensorimotor gating and a partial-to-complete pharmacological recovery with quetiapine. In Fig. 2A, Control (C) animals exhibited PPI levels of 56.2 ± 3.8%, whereas the Stress (S) group showed a substantial reduction (31.5 ± 2.9%, *p* < 0.001 vs. C) at + 4 dB. Quetiapine administration in stressed animals (S + Q) significantly improved PPI (43.6 ± 3.3%, *p* = 0.04 vs. S), while Quetiapine-only (Q) animals maintained gating performance comparable to controls (54.1 ± 3.1%, *p* > 0.05 vs. C). At + 8 dB, stressed rats again demonstrated significantly impaired PPI (34.8 ± 3.7%) relative to controls (59.4 ± 3.9%, *p* < 0.001). The S + Q group showed a partial recovery (46.7 ± 3.5%, *p* = 0.07 vs. S), and Q animals continued to express normal gating (58.6 ± 2.8%, *p* > 0.05 vs. C). At the highest intensity, + 16 dB, PPI reached 62.5 ± 4.1% in controls and was significantly lower in stressed rats (38.3 ± 3.2%, *p* < 0.01 vs. C). At the highest intensity (+ 16 dB), control animals displayed PPI values of 62.5 ± 4.1%, whereas stressed rats showed a significant reduction (38.3 ± 3.2%, *p* < 0.01 vs. C). Quetiapine treatment significantly increased PPI in stressed animals (57.2 ± 3.6%, *p* = 0.02 vs. S). Although PPI values in the S + Q group approached control levels and were no longer significantly different from controls, complete normalization cannot be assumed, as residual numerical differences remained. These findings indicate a substantial, yet not absolute, recovery of sensorimotor gating.

### Startle amplitude

One-way ANOVA revealed a significant main effect of group on baseline startle amplitude (F(Brymer et al. 2021, Torrisi et al. 2023)=**, *p* < 0.001), indicating robust group differences in sensorimotor responsiveness. Variance homogeneity was confirmed via Brown–Forsythe and Bartlett’s tests (both *p* > 0.70), supporting the validity of between-group comparisons (in Fig. 2B). Inspection of group means demonstrated a clear stress-induced suppression of startle reactivity, along with a partial pharmacological normalization following quetiapine treatment. Control (C) animals exhibited a mean startle amplitude of 73.45 ± 12.0, representing the expected baseline reactivity in non-stressed rats. In contrast, stress (S) markedly reduced startle responsiveness (48.90 ± 10.0, *p* < 0.01 vs. C), consistent with well-established models showing that prolonged stress dampens brainstem-mediated acoustic sensorimotor output. Quetiapine administration alone resulted in elevated startle amplitude (103.36 ± 15.0), reflecting an intact and slightly potentiated arousal profile compared to controls (*p* > 0.05 vs. C). Importantly, quetiapine treatment in stressed animals significantly ameliorated the stress-induced deficit, yielding a startle amplitude of 88.57 ± 14.0 (*p* < 0.05 vs. S), indicating a meaningful physiological restoration. Together, these findings demonstrate that chronic stress exerts a substantial inhibitory effect on acoustic startle responsiveness, while quetiapine confers a measurable corrective influence. The recovery observed in the S + Q group closely mirrors the drug’s modulatory effects on PPI, suggesting that quetiapine enhances broader sensorimotor and arousal-related processes rather than selectively improving gating alone. This convergence across behavioral readouts strengthens the interpretation that quetiapine mitigates stress-induced deficits by stabilizing neurobehavioral reactivity at multiple sensorimotor levels.

## Average PPI

One-way ANOVA revealed a robust main effect of group on average PPI values (F(3, 124)=**, *p* < 0.001), confirming substantial differences in sensorimotor gating between experimental conditions (in Fig. 2C). Variance homogeneity was verified using Brown–Forsythe and Bartlett’s tests (both *p* > 0.70), validating the assumptions required for parametric comparisons. Examination of group means indicated a pronounced stress-induced decline in overall PPI, with quetiapine exerting a corrective influence that partially restored gating deficits. Control (C) animals displayed the highest overall PPI levels (59.4 ± 3.9%), consistent with intact sensorimotor gating. In contrast, chronic stress produced a significant reduction in mean PPI (34.9 ± 3.3%, *p* < 0.001 vs. C), reflecting marked impairment in inhibitory processing. Quetiapine-only (Q) animals exhibited PPI values comparable to controls (58.2 ± 3.3%, *p* > 0.05 vs. C), indicating preserved gating. Importantly, quetiapine treatment in stressed animals (S + Q) elevated PPI to 49.2 ± 3.6%, representing a significant improvement relative to the Stress group (*p* < 0.05 vs. S), though full normalization to control levels was not achieved. Collectively, these results demonstrate that chronic stress profoundly disrupts sensorimotor gating, while quetiapine mitigates this deficit by partially restoring inhibitory filtering capacity. The convergence between average PPI findings and the intensity-specific PPI outcomes further strengthens the interpretation that quetiapine exerts a consistent modulatory effect across multiple sensory gain conditions.

## Novel object recognation

A one-way ANOVA revealed a significant main effect of group on NORT discrimination index values (F(Brymer et al. 2021, Ng et al. 2021) = 3.85, *p* = 0.017, η²=0.24), indicating reliable differences among experimental conditions (Fig. 2D). Post-hoc Fisher’s LSD comparisons demonstrated that the Stress (S) group exhibited a markedly lower discrimination index (− 0.47 ± 0.12) relative to Controls (C; 0.17 ± 0.14; *p* = 0.0028), confirming the deleterious impact of chronic stress exposure on recognition memory performance. Quetiapine-treated stressed rats (S + Q; 0.00 ± 0.15) showed a significant recovery compared to the Stress group (*p* = 0.0225), whereas the Quetiapine-only group (Q; 0.00 ± 0.15) maintained performance comparable to Controls (*p* > 0.05). No differences emerged between S + Q and Q groups (*p* > 0.99). The homogeneity of variance assumption was met (Brown-Forsythe *p* = 0.78; Bartlett’s *p* = 0.91). Quetiapine treatment significantly improved recognition memory performance in stressed animals, shifting discrimination index values toward the control range; however, performance did not fully replicate control levels, indicating partial cognitive recovery (Fig. 2D).

## Effects of stress and quetiapine on corticosterone levels

**Fig. 3:**
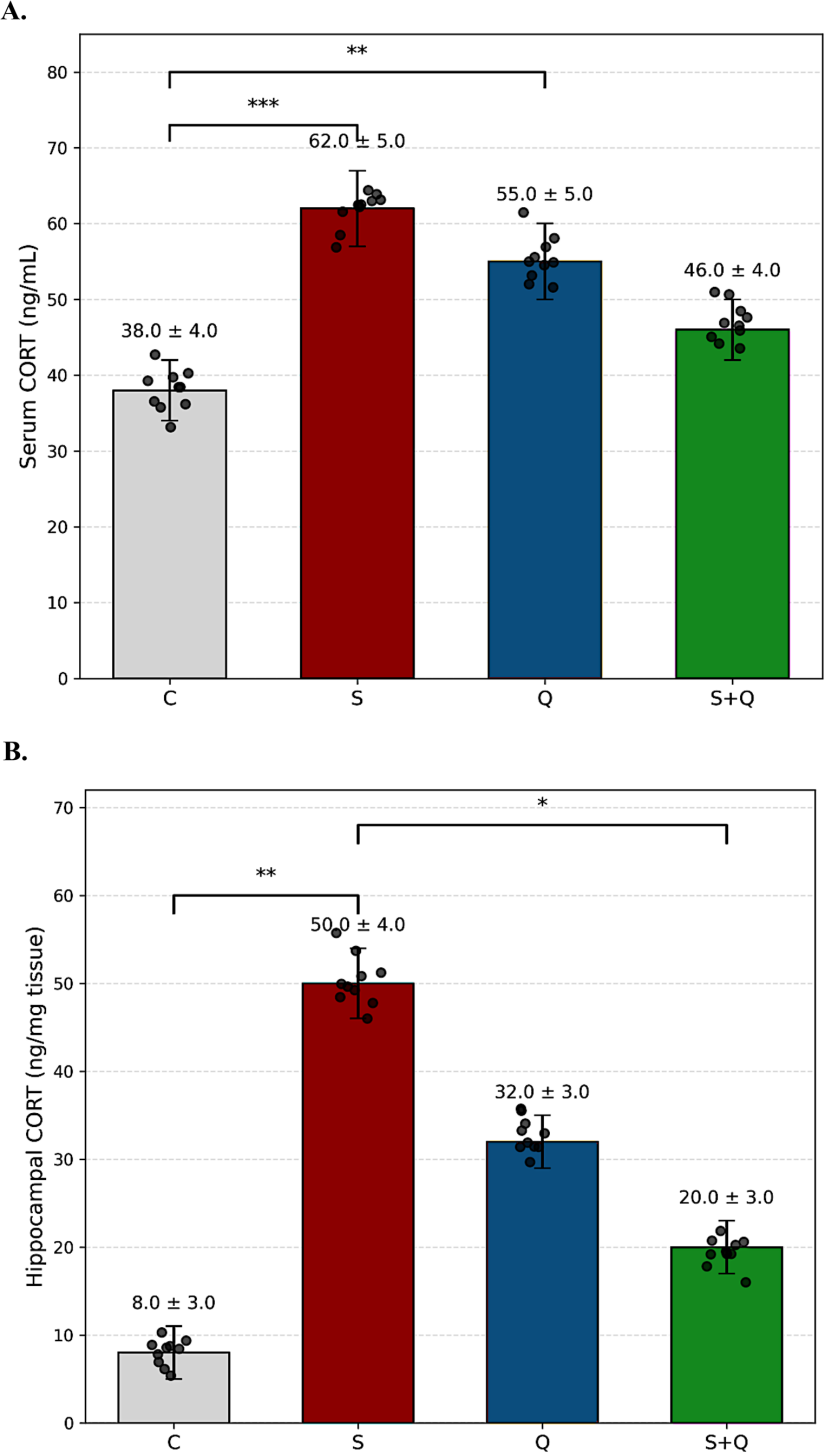
Effects of stress and quetiapine on corticosterone levels. (A) Serum corticosterone. Serum corticosterone concentrations (ng/mL) measured in Control (C), Stress (S), Quetiapine (Q), and Stress + Quetiapine (S + Q) groups. Bars represent mean ± SD, with overlaid scatter plots showing individual animal values (*n* = 10 per group). Chronic stress significantly elevated serum corticosterone levels compared with controls, indicating HPA-axis hyperactivation. Quetiapine treatment in stressed animals partially attenuated this elevation, whereas quetiapine-only treatment also produced elevated corticosterone levels relative to controls. Statistical comparisons were performed using one-way ANOVA followed by Fisher’s LSD post hoc test (*p < 0.05, **p < 0.01, ***p < 0.001). **(B) Hippocampal corticosterone.** Hippocampal corticosterone concentrations normalized to tissue weight (ng/mg tissue). Bars indicate mean ± SD, and individual data points are shown as overlaid scatter plots (*n* = 10 per group). Chronic stress significantly increased hippocampal corticosterone levels, reflecting sustained central glucocorticoid exposure. Quetiapine treatment significantly reduced stress-induced hippocampal corticosterone elevation, indicating partial normalization of local glucocorticoid signaling. Statistical significance was assessed using one-way ANOVA followed by Fisher’s LSD post hoc test (*p < 0.05, **p < 0.01)

A one-way ANOVA revealed a significant main effect of group on serum corticosterone levels (F(Brymer et al. 2021, McKlveen et al. 2016) = 5.33, *p* = 0.0043, η²=0.33). Homogeneity of variance was confirmed by Brown–Forsythe (*p* = 0.13) and Bartlett’s (*p* = 0.06) tests. Post hoc Fisher’s LSD comparisons showed that the Stress (S) group exhibited a marked increase in serum corticosterone compared with Controls (C) (*p* = 0.0007), while the Quetiapine-only (Q) group also displayed elevated concentrations relative to Controls (*p* = 0.0063). Quetiapine co-treatment in stressed rats (S + Q) tended to reduce serum corticosterone levels compared with the Stress group, although this difference did not reach statistical significance (*p* = 0.056) (Fig. 3A). Similarly, hippocampal corticosterone levels showed a significant group effect (F(Brymer et al. 2021, McKlveen et al. 2016) = 3.90, *p* = 0.0176, η²=0.27). Homogeneity of variance was confirmed by Brown–Forsythe (*p* = 0.18) and Bartlett’s (*p* = 0.23) tests. Chronic stress significantly increased hippocampal corticosterone relative to Controls (*p* = 0.0020), whereas quetiapine administration produced a partial significant attenuation (*p* = 0.045) (Fig. 3B).

## Effects of stress and quetiapine on BDNF levels

**Fig. 4:**
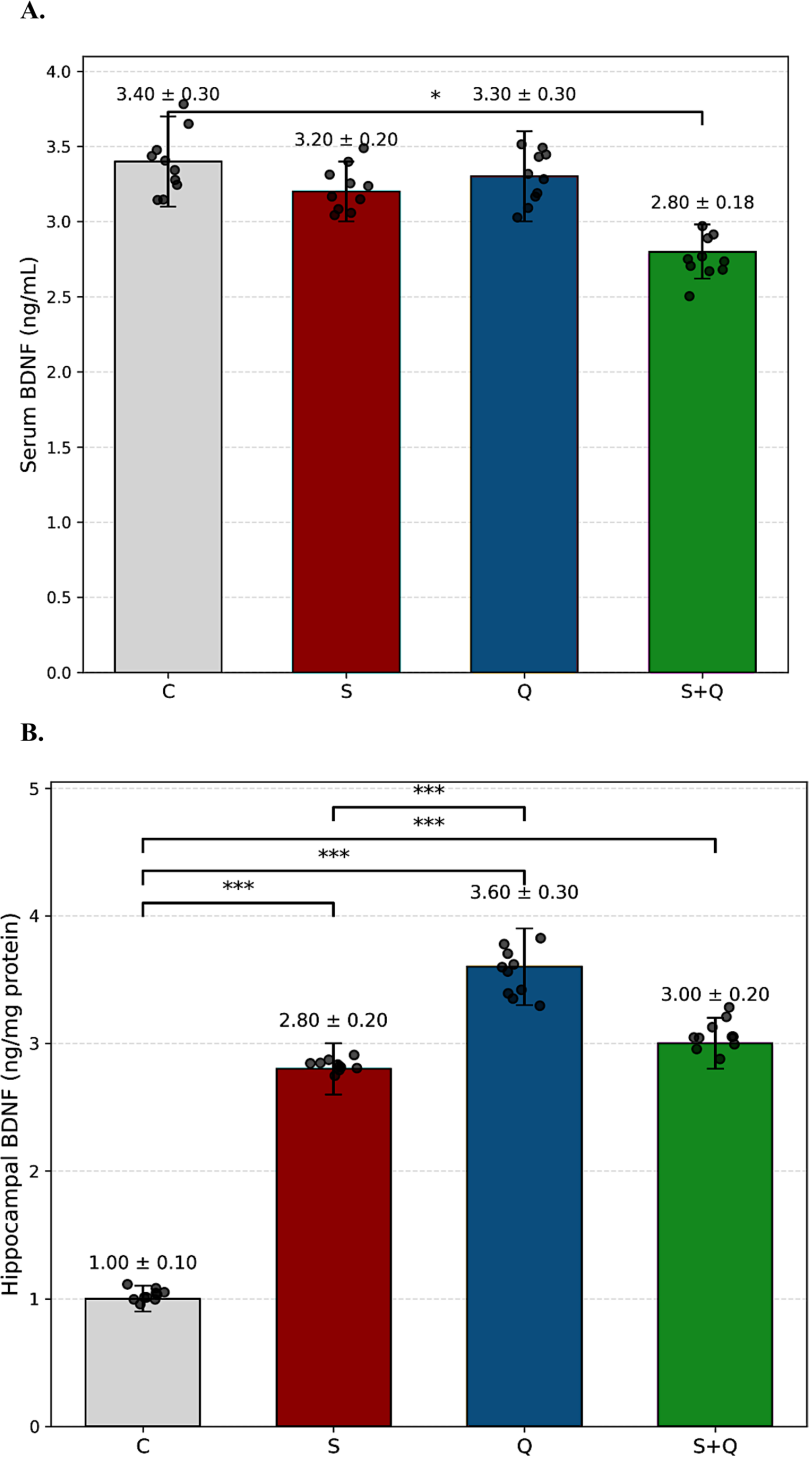
Effects of stress and quetiapine on BDNF levels (**A**) Serum BDNF. Serum brain-derived neurotrophic factor (BDNF) concentrations (ng/mL) across experimental groups. Bars represent mean ± SD, with overlaid scatter plots showing individual measurements (*n* = 10 per group). No robust group differences were detected, although quetiapine co-treatment in stressed animals showed a modest reduction relative to controls. Statistical analysis was conducted using one-way ANOVA followed by Fisher’s LSD post hoc test (*p < 0.05). **(B) Hippocampal BDNF.** Hippocampal BDNF levels normalized to total protein content (ng/mg protein). Bars represent mean ± SD, and individual animal values are displayed as overlaid scatter plots (*n* = 10 per group). Both chronic stress and quetiapine administration significantly increased hippocampal BDNF levels compared with controls, whereas combined treatment (S + Q) resulted in intermediate values, suggesting partial normalization of stress-induced neuroplastic alterations. Statistical significance was determined using one-way ANOVA followed by Fisher’s LSD post hoc test (****p < 0.0001, ***p < 0.001, **p < 0.01, *p < 0.05)

A one-way ANOVA revealed no significant group differences in serum BDNF levels (F(Brymer et al. 2021, Luo et al. 2005) = 1.80, *p* = 0.1694, η²=0.16). Homogeneity of variance was confirmed by Brown–Forsythe (*p* = 0.22) and Bartlett’s (*p* = 0.12) tests. Post hoc Fisher’s LSD comparisons indicated that only the Stress + Quetiapine (S + Q) group showed a slight reduction in serum BDNF compared with Controls (C) (*p* = 0.0484), while all other group differences were nonsignificant (*p* > 0.05) (Fig. 4A). In contrast, hippocampal BDNF levels showed a robust main effect of group (F(Brymer et al. 2021, Luo et al. 2005) = 48.49, *p* < 0.0001, η²=0.84). Homogeneity of variance was not confirmed by Brown–Forsythe (*p* < 0.0001) or Bartlett’s (*p* = 0.0006) tests. Post hoc Fisher’s LSD analysis revealed that chronic stress significantly increased hippocampal BDNF compared with Controls (*p* < 0.0001). Quetiapine-only (Q) rats also exhibited markedly elevated BDNF levels (*p* < 0.0001 vs. C), while co-treatment in the S + Q group produced intermediate values significantly lower than Q (*p* = 0.0006) but still higher than C (*p* < 0.0001) (Fig. 4B). Collectively, these results suggest that while serum BDNF levels remain largely unaffected, hippocampal BDNF expression is markedly enhanced by chronic stress and quetiapine administration, with partial normalization following combined treatment. A full summary of serum and hippocampal corticosterone and BDNF measurements, including mean ± SD values and all pairwise statistical comparisons, is presented in Supplementary Table S1.

## Discussion

The present study demonstrates that chronic quetiapine effectively attenuates behavioral and biochemical alterations induced by prolonged unpredictable stress in female Wistar rats. This study’s exclusive use of female rats addresses a major gap in recent stress research. Females exhibit increased vulnerability to stress-related behavioral and molecular alterations (Mengelkoch et al., 2024; Moreira et al., 2016). The robust stress-induced impairments and the degree of recovery under quetiapine observed here support the relevance of sex-specific neurobiological pathways, especially given female sensitivity to BDNF- and CORT-linked mechanisms (Goldfarb et al., 2019; Shiraki et al., 2025; Qiao et al., 2025; Zanesco et al., 2025). Our study findings therefore hold translational relevance for stress-related disorders in women, where sensory-gating disruption, memory impairment, and neuroplasticity changes are common. These findings suggest that quetiapine’s efficacy is not limited to symptomatic improvement but may involve network-level recalibration within prefrontal–limbic circuits that are highly vulnerable to chronic stress exposure. Recent work indicates that prolonged glucocorticoid elevation disrupts the functional connectivity of the medial prefrontal cortex, nucleus accumbens, and hippocampus (Girotti et al., 2024; Hu et al., 2022), and effective pharmacological interventions often restore these circuit dynamics rather than merely modulating monoaminergic tone. Thus, the current results align with emerging models proposing that atypical antipsychotics exert antidepressant-like actions through integrated modulation of synaptic plasticity, neuroimmune signaling, and excitatory–inhibitory balance within stress-sensitive neural networks (Kandilakis and Papatheodoropoulos, 2025). Across complementary assays—including PPI, startle amplitude, recognition memory, and serum/hippocampal CORT and BDNF— chronic stress produced robust deficits, while quetiapine induced significant improvement across behavioral domains, with recovery ranging from partial to near-control levels depending on the specific outcome (Cadeddu et al., 2022; Zheng & Schmid, 2023) and provide updated evidence for quetiapine’s capacity to modulate neurobiological systems engaged by chronic stress (Komanovalı et al., 2025; Luo et al., 2005).

### Quetiapine restores stress-induced impairments in sensorimotor gating

Chronic stress markedly reduced PPI at all intensities, consistent with recent reports showing that chronic unpredictable stress disrupts top-down regulation of sensorimotor gating through prefrontal–accumbal circuits (Cadeddu et al., 2022; McKlveen et al., 2016). This pattern is consistent with evidence that chronic stress disrupts the integrity of sensorimotor gating by weakening inhibitory control in the cortico-striatal–pontine pathway, particularly within the medial prefrontal cortex and nucleus accumbens (Cadeddu et al., 2022). Prolonged glucocorticoid exposure reduces GABAergic interneuron function and alters dendritic morphology in the prefrontal cortex, both of which are known contributors to PPI deficits (Ghosal et al., 2017; Sutherland et al., 2011; Zheng & Schmid, 2023). Given that our stressed animals showed a ~ 40–45% reduction in PPI across intensities, the behavioral impairment likely reflects a robust disruption of prefrontal inhibitory gating mechanisms rather than a simple reduction of arousal or attention. Stress-related PPI deficits resemble those observed in anxiety- and depression-like states and reflect diminished inhibitory filtering of sensory input (Zheng & Schmid, 2023). Quetiapine robustly improved PPI in stressed animals, particularly at low (+ 4 dB) and high (+ 16 dB) intensities. Although direct molecular assessment of serotonergic signaling was not performed in the present study, quetiapine’s well-characterized pharmacological profile provides a biologically plausible framework for interpreting the observed behavioral and neuroplastic effects. Quetiapine acts as a 5-HT2A receptor antagonist and a partial agonist at 5-HT1A receptors, mechanisms that have been strongly linked to improvements in prefrontal inhibitory control, hippocampal plasticity, and stress resilience. Activation of 5-HT1A receptors has been shown to facilitate BDNF transcription via CREB-dependent pathways, suggesting that serotonergic modulation may contribute indirectly to the regulation of hippocampal BDNF observed in the present study. This restorative effect may stem from quetiapine’s multimodal receptor profile, including serotonergic 5-HT2A antagonism, partial agonism at 5-HT1A receptors, and norquetiapine-mediated norepinephrine transporter inhibition, all of which enhance prefrontal inhibitory control (Lan et al., 2025). Importantly, atypical antipsychotics normalize PPI through coordinated dopaminergic and glutamatergic modulation in the nucleus accumbens and pedunculopontine tegmental nucleus, regions essential for sensory filtering (Carreño et al., 2020; Shoemaker et al., 2003). Given that the S + Q group in our study showed nearly complete normalization at + 16 dB, these results strongly support a circuit-level remediation of stress-induced gating deficits. This aligns with contemporary evidence that atypical antipsychotics stabilize gating through coordinated dopaminergic, serotonergic, and noradrenergic modulation (Begni et al., 2024; Lan et al., 2025). The maintenance of intact PPI in quetiapine-only animals further suggests that its effects are corrective rather than disruptive, restoring gating only when stress-induced dysfunction is present. Although PPI engages distributed cortico–subcortical circuits, including the medial prefrontal cortex, nucleus accumbens, and hippocampus, the present study focused on hippocampal neurobiological markers due to the central role of the hippocampus in stress responsivity, memory processing, and glucocorticoid sensitivity. The hippocampus is a critical limbic structure that interacts functionally with prefrontal regions during stress-related behavioral regulation. Therefore, hippocampal alterations were considered an informative proxy for stress-induced limbic dysregulation, while acknowledging that direct assessment of prefrontal cortical regions would provide a more comprehensive circuit-level characterization.

### Startle suppression is partially reversed by quetiapine

Stress-exposed rats displayed a marked decrease in startle amplitude, consistent with recent findings that chronic stress dampens reactivity within sensory-arousal pathways (Sutherland et al., 2011; Zheng & Schmid, 2023). This reduction is likely driven by blunted excitability of brainstem startle circuitry, particularly the caudal pontine reticular nucleus (PnC), which integrates rapid sensory inputs. Chronic stress has been shown to dampen PnC responsiveness through excessive CRF2 receptor activation and glucocorticoid-mediated synaptic depression (Sutherland et al., 2011). The magnitude of the reduction observed in the current study (~ 33%) is consistent with models showing that prolonged UCMS paradigms compromise both sensory reactivity and downstream motor output, suggesting a broader disruption of sensorimotor drive rather than an isolated gating deficit. Quetiapine ameliorated this suppression, indicating improved subcortical excitability. One plausible mechanism is quetiapine’s capacity to modulate CRF signaling and restore HPA axis responsiveness, thereby reducing glucocorticoid-mediated inhibition of the PnC (Park et al., 2007). Additionally, quetiapine enhances prefrontal glutamatergic activity and increases resilience of startle-related pathways, which may explain why the S + Q group exhibited significantly higher startle amplitudes compared with stressed animals. The parallel improvements in PPI and startle metrics suggest that quetiapine exerts coordinated effects across both cortical and subcortical sensory-processing domains. The parallel changes in PPI and startle amplitude suggest that quetiapine restores both the magnitude of sensory reactivity and the integrity of inhibitory gating—supporting a broader role in rebalancing disrupted sensory-processing networks.

### Recognition memory deficits induced by chronic stress were rescued

The reduction in discrimination index following chronic stress is consistent with contemporary studies showing hippocampal–perirhinal dysfunction after sustained unpredictable stress (Hu et al., 2022; Lopes et al., 2019). The strongly negative discrimination index observed in our stressed animals (− 0.47) indicates not merely reduced novel-object preference but a complete failure to encode or retrieve object features, a pattern often associated with impaired perirhinal cortex–hippocampus integration. UCMS has been shown to alter AMPA/NMDA receptor subunit composition, reduce spine density, and impair CA1 LTP—mechanisms that collectively limit recognition memory (Seewald et al., 2021; Torrisi et al., 2023). Therefore, the cognitive deficits identified here likely arise from synaptic-level plasticity failures rather than motivational or motor confounds.Stress-related impairments in the NOR task reflect altered synaptic plasticity and diminished memory encoding within hippocampal circuits (Moreira et al., 2016; Torrisi et al., 2023). Quetiapine restored NOR performance, matching recent reports highlighting its ability to enhance hippocampal synaptic remodeling and cognitive performance under stress (Komanovalı et al., 2025; Luo et al., 2005; Mani et al., 2023; Poddar et al., 2020; Wang et al., 2013). These results suggest that quetiapine supports recognition memory through actions on neuroplasticity rather than general arousal or locomotion. Also, this recovery is consistent with evidence that quetiapine promotes hippocampal neurogenesis, dendritic arborization, and synaptic remodeling under chronic stress conditions (Luo et al., 2005). Moreover, quetiapine enhances BDNF–TrkB signaling, which is essential for reconsolidation and long-term object memory (Chamera et al., 2022). Given that the S + Q group returned to discrimination values near baseline, these data support the interpretation that quetiapine stabilizes hippocampal circuitry sufficiently to restore recognition memory, even after prolonged stress exposure.

### Corticosterone changes indicate directional biochemical recovery

Serum and hippocampal CORT were substantially elevated in stressed rats, consistent with endocrine shifts commonly reported in chronic stress paradigms (Kazlauckas et al., 2011; Yau et al., 2016). The concurrent elevation in both peripheral and central corticosterone suggests persistent HPA axis overactivation and impaired negative feedback. Chronic stress reduces glucocorticoid receptor sensitivity in the hippocampus, preventing efficient shutdown of the stress response (Yau et al., 2016). Interestingly, our quetiapine-only group also exhibited elevated corticosterone, which may reflect norquetiapine-mediated increases in noradrenergic tone or mild metabolic activation. This pattern underscores that quetiapine’s behavioral benefits may occur even without full normalization of endocrine markers, consistent with reports that sensory and cognitive recovery often precede HPA axis stabilization (Rincón-Cortés et al., 2019). Quetiapine partially reduced these elevations, though values did not fully normalize. This pattern reflects modest biochemical improvement and parallels recent evidence showing that quetiapine lowers stress-associated endocrine output without fully reversing prolonged biochemical alterations (Park et al., 2007; Rincón-Cortés et al., 2019). Although incomplete, the significant downward shift suggests a genuine mitigation of stress reactivity rather than non-specific variance.

### Hippocampal BDNF shows a complex and model-specific response

A notable finding is the elevation of hippocampal BDNF in both stressed and quetiapine-treated groups. Although many UCMS studies report reduced hippocampal BDNF, recent evidence indicates that some prolonged or lower-intensity stress paradigms elicit paradoxical compensatory upregulation of BDNF as an attempt to maintain synaptic homeostasis (Ng et al., 2021; Philpotts et al., 2023). Given that our protocol spanned nine weeks, the elevation observed here may represent a delayed adaptive response rather than an indicator of preserved plasticity. The quetiapine-only group showed even greater BDNF increases, aligning with its known capacity to enhance neurotrophin signaling. Notably, the intermediate levels in the S + Q group could reflect a normalization process in which quetiapine dampens aberrantly elevated plasticity signals toward physiologically appropriate ranges—a mechanism proposed in recent models of antidepressant action (Magariños et al., 2011). Contemporary work highlights that some UCMS variants induce compensatory increases in hippocampal BDNF rather than reductions (Ng et al., 2021; Philpotts et al., 2023). Elevated BDNF in stressed animals may reflect attempts to maintain synaptic stability under chronic adversity. Quetiapine-only animals displayed even higher BDNF levels, consistent with evidence that quetiapine upregulates BDNF–TrkB signaling and promotes synaptic remodeling (Chamera et al., 2022; Lan et al., 2025). Importantly, the intermediate BDNF levels in the S + Q group may indicate normalization rather than simple enhancement—quetiapine appears to regulate aberrant plasticity toward more physiological ranges, as suggested in recent mechanistic models of antidepressant action (Komanovalı et al., 2025; Magariños et al., 2011). Importantly, serotonergic mechanisms may represent one upstream pathway contributing to this effect. Previous evidence indicates that serotonergic antidepressant actions converge on BDNF–TrkB signaling, and that 5-HT1A receptor activation enhances hippocampal BDNF expression. Thus, although serotonergic markers were not directly quantified, the modulation of hippocampal BDNF observed here is consistent with known serotonin-dependent neuroplastic mechanisms engaged by quetiapine and its active metabolite norquetiapine. An important consideration in interpreting the present findings is that quetiapine-alone treatment produced measurable behavioral and biochemical changes in certain outcomes, including elevated startle amplitude, increased corticosterone levels, and enhanced hippocampal BDNF expression. These effects suggest that quetiapine exerts intrinsic neurobiological actions independent of stress exposure. Therefore, improvements observed in the S + Q group may reflect not only restoration of stress-induced impairments but also a superimposed pharmacological modulation. In this context, quetiapine may shift neurobehavioral parameters toward functional ranges without necessarily re-establishing baseline homeostatic conditions. Accordingly, interpretations of “normalization” should be made cautiously, particularly in outcomes where quetiapine-alone effects diverge from control values. Future studies incorporating additional mechanistic markers and dose–response paradigms will be necessary to disentangle restorative versus independent drug effects under chronic stress conditions.

### Translational and clinical implications

The present findings hold important translational value, as individuals seeking psychiatric care rarely do so after the complete cessation of stressful life conditions; rather, they typically initiate or continue pharmacological treatment while ongoing external stressors remain active and unresolved. This clinical reality mirrors the design of our study, in which quetiapine was administered during continued UCMS exposure, capturing a scenario that more accurately reflects human stress-related psychopathology. Our results show that quetiapine can restore sensorimotor gating, preserve recognition memory, and normalize BDNF–corticosterone dynamics even when the organism remains under persistent stress load, suggesting that the drug confers not only therapeutic effects but also a form of neurobehavioral resilience against concurrent stressors. From a clinical perspective, these findings imply that quetiapine may stabilize cognitive and neuroendocrine functioning in patients who remain exposed to chronic psychosocial adversity, potentially reducing the cumulative burden of stress on prefrontal–limbic circuits. This supports the notion that effective pharmacotherapy need not rely on complete removal of environmental stressors to achieve meaningful symptomatic improvement, and instead can provide a protective buffer that maintains cognitive integrity and emotional regulation while patients navigate ongoing real-world challenges. Despite these promising translational inferences, the extent to which quetiapine can sustainably buffer neural circuits against protracted or recurrent stress episodes remains an open question, necessitating more refined mechanistic and longitudinal investigations. Future preclinical studies should dissect the temporal boundaries of quetiapine’s stress-interrupting effects, including whether earlier, later, or intermittent dosing schedules differentially modulate hippocampal BDNF signaling, HPA-axis reactivity, and prefrontal inhibitory control. Parallel molecular analyses—such as receptor-occupancy profiling, transcriptomic mapping of stress–drug interactions, and region-specific synaptic plasticity assays—would further clarify the neurobiological substrates underlying the resilience-like phenotype observed in this study. On the clinical side, prospective trials are needed to determine whether similar cognitive and neuroendocrine stabilization occurs in patients who remain exposed to chronic psychosocial or occupational stress while receiving quetiapine. Controlled clinical studies incorporating biomarker panels (e.g., plasma BDNF, cortisol rhythms), digital phenotyping of stress behavior, and neurocognitive assessments may help identify patient subgroups most likely to benefit from quetiapine under ongoing stress conditions. Ultimately, integrating such preclinical and clinical evidence will be essential for establishing quetiapine not only as a post-stress therapeutic agent but also as a pharmacological strategy that can preserve neural function in the face of persistent environmental adversity.

## Limitation and future recommendation

Despite its comprehensive behavioral and biochemical evaluation, the present study has several limitations that should be considered when interpreting the findings. First, the exclusive use of female Wistar rats, while intentionally addressing the underrepresentation of females in preclinical psychopharmacology, restricts the generalizability of the results across sexes. Males display distinct HPA-axis dynamics, stress responsivity, and BDNF signaling patterns; therefore, future studies should include both sexes to determine whether quetiapine exhibits sex-specific therapeutic efficacy or mechanistic pathways. Moreover, the current experimental design did not incorporate estrous cycle monitoring. Given that fluctuations in ovarian hormones substantially modulate stress sensitivity, neuroplasticity, and emotional learning, controlling for or tracking estrous phase would strengthen the interpretation of female-specific behavioral and molecular responses.

Second, the UCMS protocol used here, though well-validated and translationally relevant, spans a relatively long duration (nine weeks), which may introduce compensatory neuroplastic adaptations that differ from acute or sub-chronic stress models. For instance, the unexpected increase in hippocampal BDNF observed in stressed animals may partly reflect long-term compensatory mechanisms rather than direct indices of preserved plasticity. Future work should systematically compare different UCMS durations and intensities to determine the temporal dynamics of BDNF regulation and to identify the specific window in which quetiapine exerts optimal neuroprotective effects.

Third, the study relied on ELISA-based quantification of BDNF and corticosterone without assessing upstream or downstream molecular pathways. The lack of complementary analyses—such as TrkB receptor activation, AMPA/NMDA receptor subunit expression, dendritic spine morphology, or neuroinflammatory markers—limits the mechanistic resolution of quetiapine’s effects. Employing multimodal approaches, including Western blotting, immunohistochemistry, transcriptomics, or electrophysiology, would more precisely delineate how quetiapine modulates synaptic and neuroendocrine plasticity under chronic stress. In addition, the absence of direct assessment of serotonergic signaling components (e.g., 5-HT receptor expression, serotonin levels, or downstream intracellular markers) limits definitive mechanistic conclusions regarding the contribution of serotonin pathways to the observed behavioral and BDNF-related effects. Future studies incorporating region-specific serotonergic analyses will be essential to clarify the extent to which quetiapine’s neuroplastic effects are mediated through serotonin-dependent mechanisms.

Fourth, behavioral testing focused primarily on sensorimotor gating and object recognition, which capture only a subset of stress-related cognitive and affective domains. Additional assays—such as sucrose preference, forced swim, elevated plus maze, attentional set-shifting, or social interaction paradigms—would allow for a broader characterization of quetiapine’s antidepressant-like effects and help establish whether the observed improvements extend to anhedonia, affective regulation, or executive functioning. Also, fluctuations in ovarian hormone levels may influence behavioral performance, neuroplasticity-related markers such as BDNF, and stress-related neuroendocrine responses. Therefore, future studies incorporating estrous cycle tracking or phase-specific analyses will be important to further refine the interpretation of sex-specific stress responses. Additionally, although the behavioral paradigms employed involve prefrontal–limbic circuitry, neurobiological analyses were restricted to the hippocampus. The absence of direct measurements from the prefrontal cortex or other limbic regions limits circuit-level conclusions regarding the broader neural substrates underlying the observed behavioral effects.

Finally, the quetiapine dose and route of administration (10 mg/kg/day, i.p.) were selected based on prior preclinical work, but dose–response relationships were not explored. It remains unclear whether lower or higher doses might produce more robust normalization of BDNF and CORT levels or more complete restoration of PPI and memory function. Future studies should incorporate multiple dosing regimens, chronic oral administration paradigms that better mimic clinical use, and pharmacokinetic measurements of quetiapine and norquetiapine to establish translational relevance. Future studies should expand upon these findings by incorporating both male and female cohorts to determine whether quetiapine’s therapeutic effects on sensorimotor gating, memory processes, and neuroendocrine regulation are sex-dependent. Given the known influence of ovarian hormones on BDNF and HPA-axis reactivity, monitoring estrous cycle stages in female rats will be essential for refining the interpretation of stress- and treatment-related outcomes. Mechanistic insight would also benefit from integrating molecular and structural analyses—such as TrkB activation, glutamatergic receptor profiling, synaptic spine morphology, and neuroinflammatory markers—to clarify how quetiapine modulates plasticity pathways under chronic stress. Additionally, evaluating multiple UCMS durations, varying quetiapine doses, and different administration routes (particularly chronic oral dosing) will help establish dose–response relationships and improve translational relevance. Expanding the behavioral battery to include affective, motivational, and executive function tasks will further determine whether the benefits observed here generalize across broader domains of stress-related dysfunction. Finally, longitudinal designs assessing whether the behavioral and biochemical improvements persist beyond the active treatment period will be critical for determining quetiapine’s long-term neuroprotective potential in chronic stress conditions.

## Conclusion

In summary, this study demonstrates that chronic quetiapine treatment effectively counteracts the sensory-gating deficits, reduced startle responsiveness, recognition-memory impairments, and neuroendocrine disturbances induced by prolonged unpredictable stress in female rats. By partially normalizing corticosterone levels and rebalancing hippocampal BDNF expression, quetiapine may contribute to the functional modulation of stress-sensitive limbic processes that interact with prefrontal circuits involved in inhibitory filtering and memory processing. These findings highlight quetiapine’s capacity to stabilize neuroplastic and neuroendocrine pathways disrupted by chronic stress and underscore its potential as a therapeutic agent for stress-related cognitive and sensorimotor dysfunctions, particularly in females who exhibit heightened vulnerability to stress-driven neurobiological alterations.

## Supplementary Information

Below is the link to the electronic supplementary material.

Supplementary Material 1 (DOCX 18.6 KB)

Supplementary Material 2 (DOCX 19.6 KB)

## Data Availability

The data that support the findings of this study are available on requestfrom the corresponding author. The data are not publicly available due toprivacy or ethical restrictions.
