# Supplementary material for: Quetiapine ameliorates sensorimotor gating, recognition memory, and neuroendocrine plasticity in chronic stress–induced female rats
Source: Metab Brain Dis. 2026 Mar 28;41(1):69. doi: 10.1007/s11011-026-01834-8 (PMC13032972; doi:10.1007/s11011-026-01834-8)
Supplement: Supplementary file 1 — Supplementary Material 1 (DOCX 18.6 KB) [file 11011_2026_1834_MOESM1_ESM.docx]

**Supplementary Table S1-** Summary of Physiological Outcomes (Corticosterone & BDNF) Across Serum and Hippocampal Samples in Female Wistar Rats Subjected to UCMS and Quetiapine Treatment

| Parameter | Tissue | Group | Mean ± SEM | p-values | Interpretation |
| --- | --- | --- | --- | --- | --- |
| CORT | Serum | C | 38 ± 4 | Reference | Baseline serum CORT |
|  |  | S | 62 ± 5 | ***p < 0.001 vs C*** | Stress markedly elevates CORT |
|  |  | Q | 55 ± 5 | **p < 0.01 vs C,**  ns (S vs Q) | Quetiapine alone moderately increases CORT |
|  |  | S+Q | 46 ± 4 | *p = 0.056 vs S* | Partial normalization under treatment |
| CORT | Hippocampus | C | 8 ± 3 | Reference | Baseline hippocampal CORT |
|  |  | S | 50 ± 4 | **p = 0.0020 vs C** | Stress sharply elevates hippocampal CORT |
|  |  | Q | 32 ± 3 | ns vs C | Mild increase under Q |
|  |  | S+Q | 20 ± 3 | **p = 0.045 vs S** | Partial attenuation with Q |
| BDNF | Serum | C | 3.4 ± 0.3 | Reference | Normal BDNF |
|  |  | S | 3.2 ± 0.2 | ns vs C | No stress effect |
|  |  | Q | 3.3± 0.3 | ns vs C | No Q effect |
|  |  | S+Q | 2.8 ± 0.18 | **p = 0.0484 vs C** | Slight reduction under S+Q |
| BDNF | Hippocampus | C | 1.0 ± 0.1 | Reference | Baseline neurotrophic level |
|  |  | S | 2.8 ± 0.2 | **p < 0.0001 vs C** | Stress strongly increases BDNF |
|  |  | Q | 3.6 ± 0.3 | **p < 0.0001 vs C** | Quetiapine markedly elevates BDNF |
|  |  | S+Q | 3.0 ± 0.2 | **p = 0.0006 vs Q; p < 0.0001 vs C** | Intermediate level; partial normalization |

**Note.** This supplementary table summarizes the biochemical outcomes of corticosterone and BDNF assays measured in serum and hippocampal tissue across experimental groups. Data represent mean ± SD values derived from ELISA-based quantification. Statistical comparisons reflect planned contrasts for major group differences relevant to stress-induced neuroendocrine dysregulation and quetiapine-mediated recovery. Abbreviations: BDNF (Brain-Derived Neurotrophic Factor), C (Control), CORT (Corticosterone), *ELISA (Enzyme-Linked Immunosorbent Assay), HPC (Hippocampus), Q (Quetiapine), S (Stress), SEM (Standard Error of the Mean), S+Q / QS (Stress + Quetiapine), UCMS (Unpredictable Chronic Mild Stress).*
