# Supplementary material for: Quetiapine ameliorates sensorimotor gating, recognition memory, and neuroendocrine plasticity in chronic stress–induced female rats
Source: Metab Brain Dis. 2026 Mar 28;41(1):69. doi: 10.1007/s11011-026-01834-8 (PMC13032972; doi:10.1007/s11011-026-01834-8)
Supplement: Supplementary file 2 — Supplementary Material 2 (DOCX 19.6 KB) [file 11011_2026_1834_MOESM2_ESM.docx]

**Supplementary Table S2.** Behavioral Outcomes (PPI, Startle Response, and Novel Object Recognition Test)

| Behavioral Parameter | Group | Mean ± SEM / SD | p-value | Interpretation |
| --- | --- | --- | --- | --- |
| PPI +4 dB (%) | C | 56.2 ± 3.8 | Reference | Normal sensorimotor gating |
|  | S | 31.5 ± 2.9 | ***p < 0.001 vs C*** | Stress markedly impairs gating |
|  | Q | 54.1 ± 3.1 | ns vs C; ***p < 0.001 vs S*** | Quetiapine prevents stress-induced deficit |
|  | S+Q | 43.6 ± 3.3 | *p < 0.05 vs S* | Partial recovery |
| PPI +8 dB (%) | C | 59.4 ± 3.9 | Reference | Robust gating |
|  | S | 34.8 ± 3.7 | ***p < 0.001 vs C*** | Stress reduces gating |
|  | Q | 58.6 ± 2.8 | ns vs C; ***p < 0.001 vs S*** | Gating preserved under Q |
|  | S+Q | 46.7 ± 3.5 | *p < 0.05 vs S* | Treatment attenuates deficit |
| PPI +16 dB (%) | C | 62.5 ± 4.1 | Reference | Strongest gating |
|  | S | 38.3 ± 3.2 | **p < 0.01 vs C** | Stress disrupts gating |
|  | Q | 61.8 ± 3.9 | ns vs C; ***p < 0.001 vs S*** | Quetiapine maintains normal gating |
|  | S+Q | 57.2 ± 3.6 | *p < 0.05 vs S* | Near-complete recovery |
| Average PPI (%) | C | 59.4 ± 3.9 | Reference | Baseline sensorimotor gating |
|  | S | 34.9 ± 3.3 | ***p < 0.001 vs C*** | Stress markedly impairs gating |
|  | Q | 58.2 ± 3.3 | ns vs C **/** ***p < 0.001 vs S*** | Quetiapine maintains gating; significantly higher than S |
|  | S+Q | 49.2 ± 3.6 | *p < 0.05 vs S* | Partial normalization with treatment |
| Startle Amplitude (a.u.) | C | 73.45 ± 12.0 | Reference | Baseline acoustic responsiveness |
|  | S | 48.90 ± 10.0 | **p < 0.01 vs C** | Stress reduces startle amplitude |
|  | Q | 103.36 ± 15.0 | **p < 0.05 vs C; p < 0.001 vs S** | Quetiapine markedly increases startle response |
|  | S+Q | 88.57 ± 14.0 | **p < 0.01 vs S** | Strong recovery under treatment |
| NORT | C | 0.17 ± 0.14 | Reference | Normal recognition memory |
|  | S | –0.47 ± 0.12 | **p < 0.01 vs C** | Stress markedly impairs recognition |
|  | Q | 0.00 ± 0.15 | ns vs C **/** **p < 0.01 vs S** | Q preserves memory; significantly better than stress |
|  | S+Q | 0.00 ± 0.15 | *p < 0.05 vs S* | Partial recovery under treatment |

**Note.** Supplementary Table S2 summarizes behavioral outcomes including prepulse inhibition (PPI), acoustic startle amplitude, and Novel Object Recognition Test (NORT) discrimination index across experimental groups. Behavioral impairments induced by chronic stress were consistently reversed—partially or fully—by quetiapine treatment. Values are expressed as mean ± SEM. *Abbreviations: C (Control), NORT (Novel Object Recognition Test), PPI (Prepulse Inhibition), Q (Quetiapine), S+Q (Stress + Quetiapine), S (Stress), SEM (Standard Error of the Mean), SD (Standard Deviation), a.u. (arbitrary units).*
